# Improved access and care through the implementation of virtual Hallway, a consultation platform in Nova Scotia: preliminary findings from a feasibility evaluation

**DOI:** 10.1186/s43058-024-00651-3

**Published:** 2024-10-18

**Authors:** Gail Tomblin Murphy, Tara Sampalli, Prosper Koto, Caroline Chamberland-Rowe, Julia Guk, Nelson Ventura, Justin Hartlen, Daniel Rasic, Jonathan Allen, Kianna Benson, Ryan MacNeil

**Affiliations:** 1 Nova Scotia Health, 90 Lovett Lake Crt, Suite 201, B3S 0H6 Halifax, NS Canada; 2 Virtual Hallway, 5663 Nora Bernard St. Suite 200, B3K 1B6 Halifax, NS Canada

**Keywords:** Virtual specialist consultation, Virtual Hallway, Return on investment; in-person referrals avoided, Provider experience

## Abstract

**Background:**

While previous studies have examined various platforms that enable providers to connect, Virtual Hallway (VH) stands out with its unique features. The value add is that this online platform connects primary care providers and specialists for synchronous phone-based conversations and aims to reduce referrals and enhance the quality of referrals. VH allows providers to easily log in, select the required specialty, book call times, receive reminders, and have calls documented, ensuring a high connection rate. In May 2022, the provincial health authority in Nova Scotia, a Canadian province, and VH initiated a feasibility study facilitated through the Health Innovation Hub in Nova Scotia. The goal was to enable primary care providers to connect with specialists, thereby reducing wait times and unnecessary referrals, and facilitating timely access to relevant clinical direction for patients. The current evaluation assessed utilization, value for money in economic analysis, and consultation experiences.

**Methods:**

The study used post, cross-sectional, and cost-benefit study designs. We collected data through various methods, including administratively recorded utilization, theory-driven surveys, and cost data. Utilization was measured by the number of completed consults and the number of healthcare professionals using the VH platform. We analyzed the data using a combination of descriptive statistics and a cost-benefit analysis, which also involved conducting probabilistic sensitivity analysis.

**Results:**

The study found that approximately 84% of the VH consultations avoided needing in-person specialist referrals. The return on investment was 1.8 (95% CI: 0.8 to 3.0), indicating that the monetary value of the measurable benefits associated with VH exceeded the value of the resources invested. The provider experience survey revealed high satisfaction levels with VH across user groups, with 92% of specialists and 96% of primary care providers reporting being satisfied or highly satisfied with their experience. These positive indicators of provider experience were further supported by the fact that 97% of respondents agreed or strongly agreed that they intended to continue to use VH in their practice, and 97% of respondents agreed or strongly agreed that they would recommend VH to a colleague.

**Conclusions:**

The study suggests that VH was well-received by users, with high levels of satisfaction reported and a reduced need for in-person referrals. It also represented value for money. Further research could explore how the availability of virtual health services can lead to reduced utilization of healthcare resources among different groups of patients.

**Supplementary Information:**

The online version contains supplementary material available at 10.1186/s43058-024-00651-3.

Contributions to the literature

- The study provides valuable insights into the usefulness of virtual consultations.
- The quantification of the return on investment, estimated at 1.8 (95% CI: 0.8 to 3.0), and the expected reduction in in-person referrals (84%) are particularly useful in understanding the benefits of VH.
- Additionally, the report on provider experience adds to the knowledge base on the acceptability of virtual consultations.
- These findings can inform VH and other virtual consultation platform adoption decisions elsewhere in the future.

## Background

Access to specialist healthcare services and lengthy wait times are significant challenges healthcare systems face worldwide [1, 2]. These challenges are primarily due to limited resources, such as a shortage of specialist physicians, diagnostic equipment, and treatment facilities. Additionally, the growing demand for specialist services due to increasing populations, geographic disparities in the distribution of specialists, particularly in rural and remote areas, and complex referral processes contribute to the problem. Prioritization of access based on clinical urgency also results in longer wait times for patients with less urgent conditions. However, delays in accessing specialist care can worsen these patients’ health outcomes [2, 3]. Virtual care and virtual consultation are increasingly utilized to improve access to specialist care.

A systematic review conducted by Liddy et al. [4] assessed the impact of communication between primary care providers and specialist physicians through asynchronous, directed communication over a secure electronic medium. The study found that providers had positive perspectives about the new type of access, citing timely advice from specialists, good medical care, confirmation of diagnoses, and educational benefits. Patient satisfaction scores were improved in response to quick response times and avoidance of referrals. The review provided limited reporting of system and health outcomes, such as cost impacts [4].

Previous studies have examined platforms that allow providers to connect and established numerous advantages for the healthcare system and patients [5–7]. Most have demonstrated that these platforms can expedite primary care providers’ access to specialist consultations. These studies have further established that specialist guidance provided through a consultation platform enables the primary care provider to manage the patient without necessitating a face-to-face specialist referral [5–7]. By eliminating the need for in-person interaction, phone-based provider consults save valuable time and effort for providers and the entire healthcare infrastructure. The time-saving impact of these platforms is evidenced by the current literature, which indicates that wait times associated with virtual provider consults are substantially shorter than traditional referrals, with most virtual provider consult wait times averaging under three days [8, 9]. The benefits of these consults have also been shown to reduce costs and mitigate disparities in healthcare access. These consultations effectively diminish barriers, making care more accessible, acceptable, available, affordable, and suitable [10]. On average, patients and healthcare providers in Canada report high satisfaction levels with the consultations [8, 11, 12].

## Rationale—Nova Scotia context and opportunity

Canada has a healthcare system that is publicly funded and provides universal coverage for healthcare services [13]. This means eligible individuals can access such services without paying for them at the point of care. While federal principles guide healthcare, it is primarily the responsibility of the provinces and territories to administer healthcare services [13]. They are responsible for delivering services, setting priorities, and allocating resources. Primary care is usually the entry point into the healthcare system for most Canadians. Primary care providers serve as the first contact line for patients seeking medical care. They coordinate patient care and refer patients to specialists or other healthcare services when necessary [14].

Nova Scotia is one of Canada’s eastern provinces on the Atlantic coast. As of April 2023, the province had a population of approximately one million [15]. As with other provinces and territories in Canada, Nova Scotians have significant wait times to see specialists for their care. In 2022, the median wait time from referral by a GP to a specialist in Canada was 12.6 weeks; it was 37.3 weeks for Nova Scotia [16]. Coordinating consultations between busy clinicians can be inefficient using conventional communication channels.

Responding to this priority need to enhance access to specialists to reduce lengthy wait times, one of the innovations that has emerged in Nova Scotia is the Virtual Hallway (VH). Clinicians have developed this innovation, a consultation platform facilitating direct communication between primary care providers and specialists.

### Virtual Hallway (VH) implementation in the provincial health authority

In May 2022, the provincial health authority in Nova Scotia and Virtual Hallway launched a feasibility study facilitated through the Health Innovation Hub in Nova Scotia to evaluate the impact of VH as a tool for primary care providers to connect with specialists to reduce wait times, reduce unnecessary referrals and facilitate timely access to relevant clinical direction for patients.

VH is an online platform that facilitates provider-to-provider patient-focused virtual consultation via synchronous telephone conversations. To initiate a phone consult request, a requesting provider (usually a primary care provider but occasionally a specialist) logs onto the Virtual Hallway system and completes an electronic form for a patient-specific question, with an option to attach any relevant patient documents (e.g., laboratory results, images). Primary care providers submitting the request for consult can book a phone consultation with a specific specialist of their choosing. The service is offered at no cost to patients and providers, and fee-for-service specialists and family physicians are reimbursed using existing provincial billing codes. The encounter consists of a brief phone call (typically about 10 min) between the providers that occurs on a date and time specified by each provider. See Supplemental File 2 for images of the Virtual Hallway consult booking platform. At the conclusion of each phone consult, the specialist completes a consult report summarizing the advice given.

VH has unique features and adds value compared to other existing systems. It was specifically designed to enhance collaboration and communication among healthcare providers and focuses on leveraging the expertise of specialists to support primary care providers in managing patient care effectively. VH enables primary care providers to receive expert advice rapidly without scheduling formal appointments. Unlike many existing platforms, such as telehealth services, which focus on patient-doctor communication, VH seamlessly integrates into the existing workflows of healthcare providers and offers asynchronous communication options. This allows specialists to respond at their convenience, thus not disrupting their schedules. VH also reduces the need for referrals and follow-up appointments, leading to quicker patient management and decision-making. While telehealth platforms can provide quick access to care, the process of arranging and conducting consultations can be slower.

The implementation of VH was conducted in partnership with the CANHealth Network and first enabled primary care providers to access specialists in Internal Medicine, Gastroenterology and General Surgery. The list of specialty areas available for consultations was later expanded to include psychiatry, endocrinology, hematology, hepatology, pediatrics, child/adolescent psychiatry, rheumatology, neurology, infectious diseases, Ear, Nose, and Throat (ENT), orthopedics, urology, sleep medicine, dermatology, pain management, sport & exercise medicine, pediatric allergy, geriatric medicine, gynecologic oncology, medical genetics, nephrology, palliative care, pediatric neurology, plastic surgery, respirology, and vascular surgery.

The pilot implementation involved direct engagement with primary care providers to encourage the use of VH to interact with specialty areas and elicit feedback on provider experiences using the platform.

To promote the adoption and utilization of VH among primary care providers, a variety of implementation strategies were employed, guided by the Expert Recommendations for Implementing Change (ERIC) taxonomy and recommendations by Proctor et al. [17, 18]. These strategies included:

1. Educational Meetings and Materials: Educational sessions were conducted to inform providers about the features and benefits of VH. Educational materials, including user guides and FAQs, were distributed through email and on the website to facilitate understanding and ease of use.
2. Champions: Local champions within clinics were identified and trained to advocate for VH, provide peer support, and assist colleagues with onboarding and troubleshooting.
3. Audit and Feedback: A system of feedback was implemented within the platform where providers received reports on their usage of VH, including metrics on referral reduction and connection success rates, to motivate continued and effective use.
4. Reminders: Automated email and SMS reminders and alerts were utilized to prompt providers about upcoming consultations and encourage timely documentation of calls.
5. Technical Assistance: Continuous technical support and helpdesk services were offered to address any technical issues and ensure seamless operation of the VH platform.
6. Marketing strategy: VH’s marketing strategies involved updating their website to enhance online engagement and delivering two targeted webinars to increase outreach.

## Aims and objectives

The current analysis differs from and builds upon a previous independent study that examined the VH platform in Nova Scotia [3]. The main distinction is that the current study includes an explicit economic evaluation, expands on providers’ experiences with individual consultations to describe their overarching experience integrating VH into their clinical practice, and follows implementation science principles for the evaluation.

The current study had three aims. Firstly, we aimed to examine whether the implementation of VH met its intended outcomes. The outcomes included utilization, as measured by the number of completed consults and the number of healthcare professionals using the VH platform, the number of in-person referrals avoided, as well as consultation experiences. The objective was to examine service utilization and measure consultation experiences with VH. Secondly, we aimed to assesses whether VH delivers value for money and optimal resource utilization. The objective was to understand the implementation and ongoing costs associated with VH compared to the benefits gained. Finally, we aimed to identify areas for improvement in VH’s implementation.

## Methods

### Study design

In the case of aim one, we used both post and cross-sectional study designs to examine whether the implementation of VH met its intended outcomes [19, 20]. We examined utilization using a post-study design [21]. On the other hand, the cross-sectional study design involved collecting data from providers across different specialties at a single time, which allowed us to gain a broader perspective on the impact of the VH platform. In the case of aim two, we employed a cost-benefit analysis study design to evaluate the value for money. We also used a cross-sectional study design for aim three.

### Study population

The study focused on healthcare providers, such as primary care physicians and specialists, who have incorporated the VH platform into their clinical practice. To be included in the study, participants needed to have used the platform at least once. The study population comprised various specialists to ensure diverse representation.

### Theoretical frameworks and survey instruments

A structured survey was developed for the consultation and provider experience surveys. The Consolidated Framework for Implementation Research (CFIR) [22], the Quadruple Aim framework [23], and the Non-adoption, Abandonment, and challenges to the intervention’s Scale-up, Spread, and Sustainability (NASSS) framework [24] were used to design the survey questions to ensure comprehensive and relevant data collection. These frameworks guided the formulation of questions to address the study’s objectives and to capture meaningful insights from the respondents.

CFIR is a structured approach to assess and understand various factors influencing implementation outcomes [22]. It identifies barriers and facilitators to implementation, helps select appropriate strategies, and guides evaluation efforts [22]. The Quadruple Aim is a comprehensive and effective approach that prioritizes four key areas in healthcare: improving the patient experience, improving population health, reducing the costs of healthcare, and improving the work life of healthcare providers [23]. Improving the patient experience involves enhancing the quality and satisfaction of care from the patient’s perspective, ensuring that healthcare is patient-centered and responsive to individual needs and preferences [23]. Improving population health aims to enhance overall health outcomes by addressing a wide range of factors, including social determinants, prevention, and chronic disease management [23]. Reducing the costs of healthcare focuses on lowering per capita healthcare expenses while maintaining or improving care quality, aiming for more efficient and effective use of resources [23]. Improving the work life of healthcare providers emphasizes the importance of the well-being of healthcare professionals [23]. The NASSS framework was used to assess the implementation of VH. It helps to identify the factors that influenced the success or failure of VH and provides a structured approach to improving its performance. The primary care physician and specialist physician post-consultation surveys comprised a short series of closed-ended questions to capture quantitative data on the outcomes of each consultation. The provider experience survey comprised both closed-ended and open-ended questions to capture quantitative data and qualitative insights. Most of the quantitative survey questions utilized a 5-point Likert scale, with responses ranging from strongly disagree to strongly agree.

### Data collection procedures

#### Survey data

The primary care provider and specialist physician post-consultation survey data were collected through an automated pop-up on the VH platform following the completion of each consultation. The provider experience survey data were collected through an online survey distributed via Research Electronic Data Capture (REDCap), a secure, web-based application designed to support data capture for research studies. Eligible participants received an email invitation with a link to the survey. Participation was voluntary, and responses were anonymous to ensure confidentiality.

First, a post-consultation survey was administered to both the primary care provider and the specialist physician at the end of each consultation for five months to understand their satisfaction with, and the efficiencies gained through, each individual consultation. See Supplemental File 3 for the short post-consultation survey. Second, a provider experience survey, encompassing quantitative scales and open-ended qualitative questions, was administered between November and December 2022 to elicit providers’ overarching assessment of VH and their experiences integrating the platform into their clinical practice. See Supplemental File 4 for the provider experience survey. Our sampling methodology used a pragmatic approach, convenience sampling, and allowed us to collect data from diverse participants. However, we did not conduct formal sample size calculations.

#### Utilization data

The utilization data available through the VH platform covered May 2022 to January 2024. Physicians were not officially invited to join the platform but rather discovered it through word of mouth, news releases, and Virtual Hallway marketing. According to the Canadian Institute for Health Information (CIHI), at the end of 2022, there were 1,346 family medicine physicians and 1,412 specialists in Nova Scotia [21]. Utilization was measured using the number of consults completed and the number of healthcare professionals using the VH platform.

#### Data analysis

We analyzed quantitative data using descriptive statistics such as frequencies, percentages, means, medians, and standard deviations as needed. For qualitative data obtained from open-ended questions, we used thematic analysis. We coded the responses to identify common themes and patterns related to user experiences and perceived impact. Our aim for the survey results was to provide a descriptive analysis using summary statistics.

#### Economic analysis method

The economic analysis adopted an implementation-effectiveness and cost-benefit analysis (CBA) framework [25, 26]. We closely followed the 2022 version of the Consolidated Health Economic Evaluation Reporting Standards (CHEERS) guidelines for economic evaluations in our approach. The economic analysis aimed to quantify the net cost-savings, the benefit-cost ratio, and the rate of return on investment associated with the VH covered the period from May 2022 to November 2023. The economic analysis was conducted from a healthcare payer perspective, meaning that the analysis was carried out from the point of view of the entity that pays for healthcare services. This perspective would consider the costs associated with providing healthcare services to patients and the potential benefits and outcomes of those services.

CBA involves analyzing the costs and benefits of VH compared to the status quo, which refers to what would happen without VH. This analysis helps decision-makers determine whether the benefits of VH outweigh the costs [27]. In this case, the costs included implementation and ongoing costs.

The implementation costs consist of an initial fee of about $1,265 (SD: $230) per specialist group using the platform. VH’s implementation costs were offered as a bundled package, covering the cost of acquiring the license and the associated services. The bundle also included costs associated with marketing, email and text message reminders, and the educational materials provided. The initial fee may vary depending on whether there was a front-facing customization. Additionally, Nova Scotia Health incurs an ongoing monthly license fee of around $10,000. There were no additional training and sensitization-related costs. The precise costs can fluctuate significantly based on the implementation’s specific requirements [28].

The implementation costs were obtained from project documents, while the primary care provider and specialist costs were sourced from the Nova Scotia Physicians’ Manual. We included a 7% opportunity cost of capital in the analysis [29, 30]. The opportunity cost of capital measures the economic value of funds foregone when choosing one investment over another. It includes the time value of money and a risk premium, accounting for uncertainties and potential fluctuations in returns. Considering the opportunity cost of capital is crucial for determining the net present value (NPV) of a project, which evaluates whether it will generate sufficient returns compared to its costs. A positive NPV at a discounted rate of 7% justifies the investment [29, 30]. Factoring in the opportunity cost of capital ensures that the project is not only feasible in the short term but also sustainable in the long term, accounting for economic fluctuations and long-term trends.

In Canada, the healthcare system is publicly funded. Medical Service Units (MSUs) are a standardized measurement used to quantify the value of medical services provided by healthcare professionals, especially for billing and compensation purposes. These units are essential in the fee-for-service model, where healthcare providers are paid based on the number and complexity of services they provide. MSUs standardize the measurement of complexity, time, skill, and resources required for various medical procedures and services. Each service or procedure is assigned a specific number of MSUs. Physicians and other healthcare providers use MSUs to bill provincial health insurance plans for the services they provide to patients. The total payment for a service is calculated by multiplying the number of MSUs by the assigned monetary value for each unit.

The benefits included the monetary value of in-person consultations avoided. The cost avoided per consult consisted of two components. In the first component, the per unit cost of using VH comprised the ‘Family physician-to-specialist phone call (family physician requesting advice from a specialist),’ health service code CONS 03.09 L with an MSU of 11.5 and the Specialist-to-physician (or nurse practitioner, NP) phone call (specialist providing advice to referring provider), health service code VIST 03.09 K with an MSU of 25. We multiplied the MSU by a rate per MSU of $2.68. The first component is paid even with VH. In the second component, we used the specialist fee codes, assuming comprehensive consultation, to measure the costs to the system if the avoided in-person referrals had taken place. The difference between the second and first cost components described above was the net cost per in-person consult avoided. We measured all costs in 2022 Canadian dollars.

The Canada Health Act is a crucial law that governs Canada’s healthcare system, ensuring that all insured individuals have access to medically necessary healthcare and essential hospital and physician services, regardless of their financial situation [31]. The federal government sets criteria for the provinces and territories, including Nova Scotia, through the Canada Health Transfer, which helps to keep healthcare costs in line with actual costs, as opposed to prices or charges [31]. Therefore, healthcare costs used in the analysis closely approximate the costs incurred, excluding vendor-related costs.

#### Sensitivity analysis – economic analysis

In a sensitivity analysis, we are interested in how changes in the parameters used impact our results [32, 33]. We performed two types of sensitivity analysis: a one-way sensitivity analysis and a probabilistic sensitivity analysis (PSA) [32, 33]. A one-way sensitivity analysis is used in economic analysis to determine a single variable’s effect on a model’s outcome. In this type of analysis, only one variable is changed, while all other variables are kept constant to observe the impact on the overall result. One-way sensitivity analysis aims to identify how sensitive the model is to variations in a particular variable [32, 33]. This analysis helps decision-makers understand each variable’s importance and how changes in the variable could affect the outcome. We evaluated how doubling the implementation, and ongoing costs would impact results in a one-way sensitivity analysis.

We performed a PSA to evaluate the uncertainties around the estimates for the economic analysis [33]. A PSA adds an additional layer of understanding to an economic analysis. It helps assess how simultaneous changes in the model’s parameters affect the outputs. It provides a way to examine how the analysis results may change based on uncertain factors, such as the accuracy of the data used, changes in trends, and the assumptions made in the analysis. To conduct the PSA, we identified the variables likely to significantly impact the analysis’s outcome based on expert opinion. Where there was no data on the parameters’ variability, we assumed a standard deviation of 10% of the mean in the PSA. Then, based on the literature, we assigned probability distributions to these variables. We fitted costs and in-person visits avoided to a gamma distribution and proportions to a beta distribution. Next, we used a Monte Carlo Simulation to run multiple (1000) analysis iterations, with different values drawn randomly from the assigned probability distributions for each variable. This allowed us to observe the possible outcomes’ range. We used the PSA results to calculate the 95% confidence intervals around the estimated costs and benefits of the intervention. Confidence intervals are a range of values that are likely to include the true value of the estimate with a certain level of probability.

## Results

### Utilization

At the end of January 2024, there were 1016 healthcare professionals on the VH platform, of which 873 were primary healthcare providers (86%) and 143 were specialists (14%) (Table 1). The 837 primary care providers on the VH platform represent approximately 65% (837/1,346) of the total primary care providers at in Nova Scotia as at the end of 2022. The number of specialists on the platform represent 10% of the total number of specialists in the province as at the end of 2022. There were 6,072 consultations completed on the VH platform. The top 10 most active specialties accounted for 74% of the total consults (4,511/6,072). The top 10 were defined as specialties with a minimum of 100 consultations. Internal Medicine consults accounted for a majority of the consults at 22% (1,318/6,072), followed by Psychiatry with 17% (1,020/6,072) of consults (Table 2).

**Table 1:** Cumulative number of healthcare professionals on the VH platform, January 2024 source: based on utilization data from the VH platform

Healthcare Professional	Count	Share (%)
Number of primary care providers on the Virtual Hallway platform	873	86%
Number of specialists on the Virtual Hallway platform	143	14%
**Total**	1016	100%

**Table 2:** Top 10 VH platform consultations by a specialist, January 2024

Specialty	Share (%)	Number of consults
Internal medicine	21.7%	1318
Psychiatry	16.8%	1020
OB/GYN	7.1%	431
Endocrinology	6.3%	383
Hematology	5.3%	322
Hepatology	4.6%	279
Pediatrics	3.9%	237
Rheumatology	4.0%	243
General Surgery	1.7%	103
Infectious Diseases	2.9%	176

The top ten are defined as Specialists with a minimum of 100 consultations

### Economic analysis results

According to primary care providers who completed the post-consultation survey, 84% of the total VH consults avoided needing an in-person referral. This was used to measure the number of in-person consultations avoided per specialist by multiplying the number of VH consultations for each specialist by 0.84. There were an estimated 5,101 referrals avoided.

The estimated implementation and ongoing cost was $145,020, with a 95% confidence interval (CI) of $100,014 to $203,922. Thus, we can be 95% confident that the true implementation and ongoing costs lie between $100,014 and $203,922. The cumulative net benefit (benefits minus implementation and ongoing costs) was $244,419 (95% CI: $164,996 to $325,643), meaning the expected monetary benefits exceed the costs, and we can be 95% confident the true net benefit ranges from $164,996 to $325,643 (Table 3). The return on investment (ROI) was 1.8 (95% CI: 0.8 to 3.0). The positive ROI means that the monetary benefits associated with VH exceed the costs. The benefit-cost ratio was 2.8 (95% CI: 1.8 to 4.0) (Table 3). See Table 3 for additional results. From the sensitivity analysis, when the costs were doubled, the ROI decreased from 1.8 to 0.4 (95% CI: -0.2 to 1.1). Based on all the results, the monetary value of the measurable benefits associated with VH exceeds the value of the resources invested.

**Table 3:** Net benefits associated with virtual Hallway (VH)

Indicator	Reference case		Sensitivity analysis: 100% increase in VH costs VH vs. Do-nothing Mean, (95% CI)
	Do nothing	VH vs. Do-nothing Mean, (95% CI)	
Implementation and ongoing costs	$-	$145,020 ($100,014 to $203, 922)	$290,513 ($190,883 to $418,463)
In-person referral costs avoided	$-	$389,439 ($331,417 to $451,226)	$387,977 ($299,124 to $467,897)
Benefit-cost ratio	-	2.8 (1.8 to 4.0)	1.4 (0.8 to 2.1)
Cumulative net benefits	$-	$244,419 ($164,996 to $325,643)	$97,464 (-$67,228 to $228,800)
Return on investment (ROI)	-	1.8 (0.8 to 3.0)	0.4 (-0.2 to 1.1)

By fitting key parameters to appropriate probability distributions and running the model 1000 times, we generated a distribution of outputs that helped us quantify the uncertainty around the results. We used this approach to ensure the results were reliable and accurate, considering the variations and uncertainties in the parameters. To fit the costs and in-person visits avoided, we used a gamma distribution, and for the proportions, we used a beta distribution

### Post-consultation survey results

**Fig. 1:**
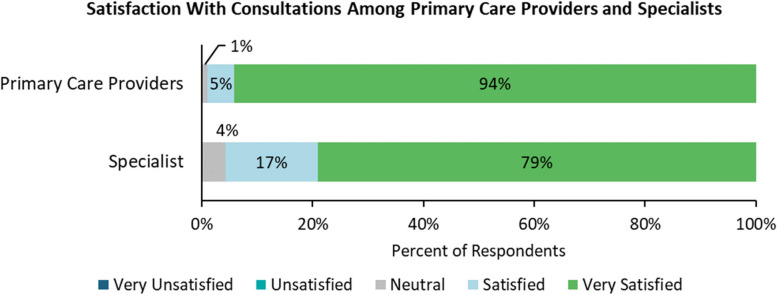
Satisfaction with consultations among primary care providers and specialists

The post-consultation survey targeted at primary care providers elicited 608 responses. Of these responses, 99%, as depicted in Fig. 1, were satisfied or very satisfied with the associated VH consultation. The post-consultation survey targeted at specialists elicited 653 responses. Of these responses, 96% of specialists were satisfied or very satisfied with the associated VH consultation.

**Fig. 2:**
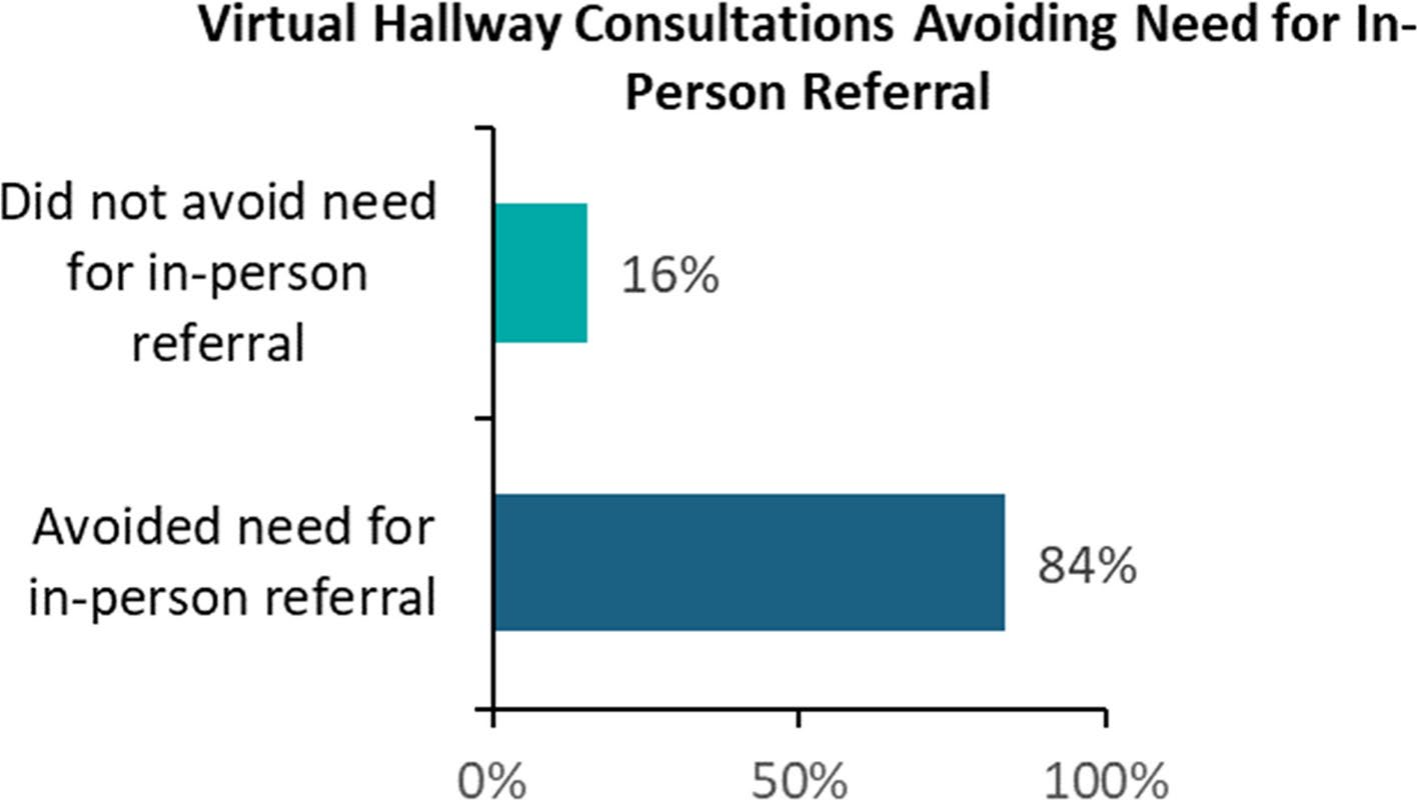
Percent of VH consultations avoiding need for in-person referral

As is depicted in Fig. 2, of the 608 VH consultations associated with a survey response from a primary care provider, 84% were reported to have avoided the need for an in-person referral, resulting in 511 fewer referrals.

Of the 97 VH consultations with a corresponding survey response that did not avoid the need for an in-person referral, 89% were never intended to avoid a referral, 89% were reported to have improved the quality of the resulting referral, and 90% were reported to have improved the associated patient’s care while they wait for an in-person referral. Furthermore, for all consultations intended to avoid a referral but did not, the associated provider indicated that the consultation had improved the quality of their referral and the patient’s care.

**Fig. 3:**
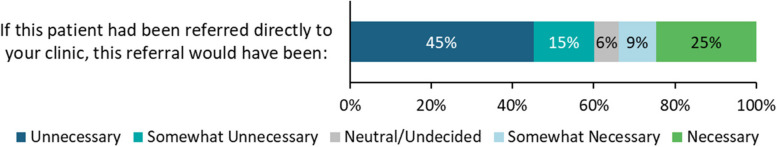
Specialists’ assessment of the necessity of direct referrals of patients associated with VH consultations

Through the survey, specialists reported on the necessity of the referral if the patient had been directly referred to their clinic for 638 VH consultations. As illustrated in Fig. 3, specialists reported that the direct referral of 55% of patients associated with VH consultations would have been either unnecessary or somewhat unnecessary. In contrast, the direct referral of 40% of patients would have been somewhat necessary or necessary. While this indicates that VH consultations avoid unnecessary referrals, these data also suggest that specialists considered direct referral an appropriate alternative to VH consultations for many patients.

### Provider experience survey

**Fig. 4:**
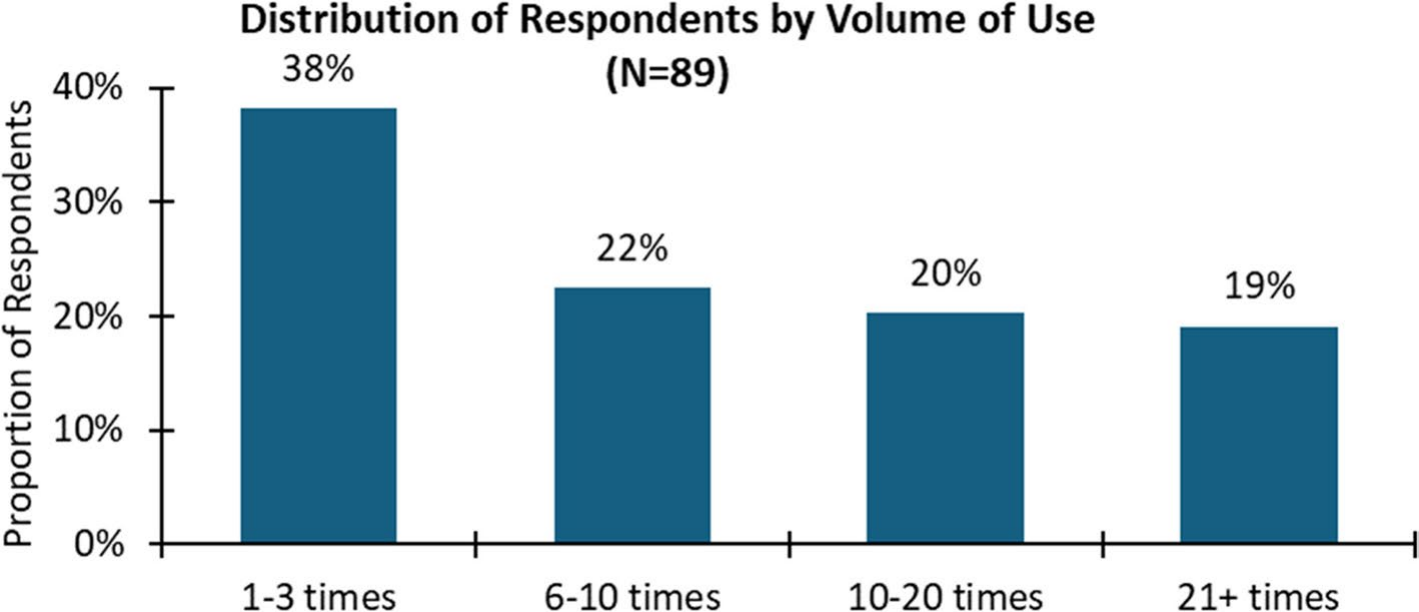
Distribution of respondents by volume of use

The majority of respondents had used the platform between 1 and 10 times, with 34 (out of 89) respondents (38%) reporting having used the platform between 1 and 5 times and 20 respondents (22%) reporting having used the platform between 6 and 10 times (Fig. 4). There were, however, a considerable number of respondents who appeared to have integrated the platform into their practice, with 18 respondents (20%) having used the platform between 10 and 20 times and 17 respondents (19%) reporting having used the platform for consultations more than 20 times (Fig. 4).

**Fig. 5:**
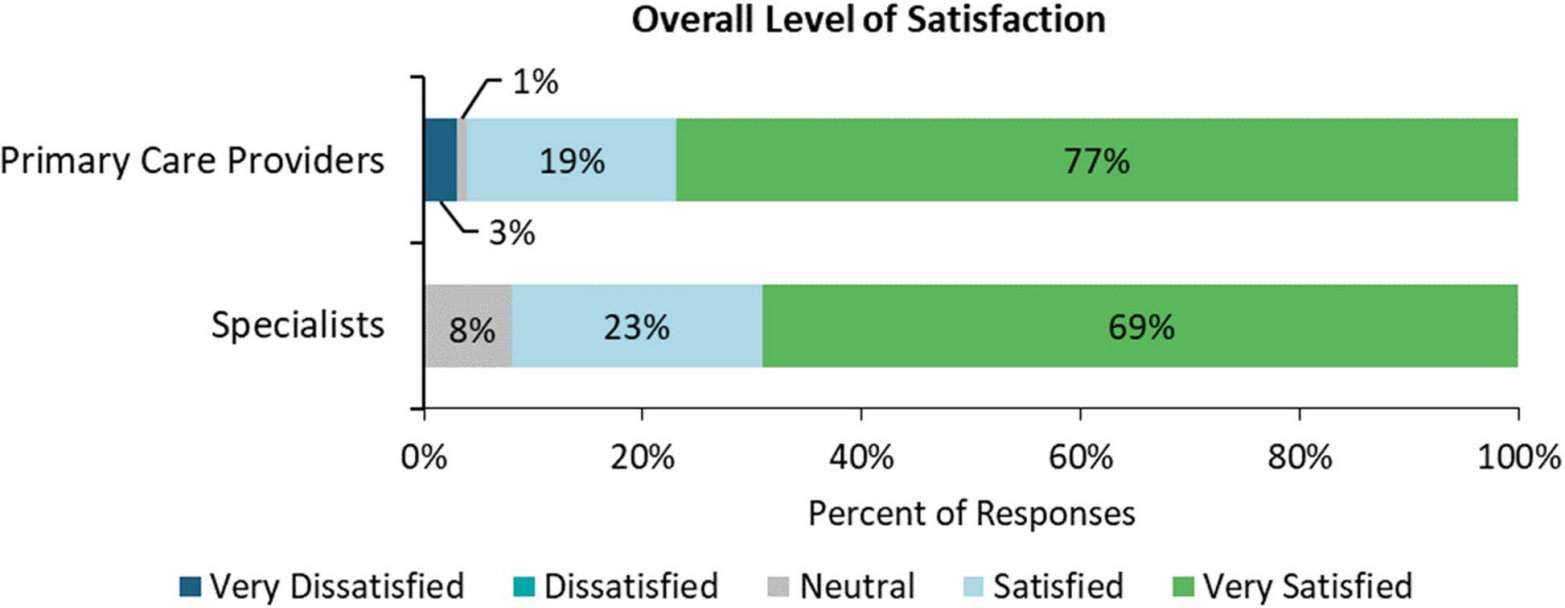
Overall level of satisfaction

As illustrated in Fig. 5, the results of the provider experience survey showed that primary care providers displayed slightly higher levels of satisfaction with their experience using VH. 92% of specialists reported being satisfied or highly satisfied with their experience, and 96% of primary care providers fell into this category. Despite this small difference, these results indicate high levels of satisfaction with VH across user groups.

These positive indicators of provider experience are reinforced by the fact that 97% of respondents agreed or strongly agreed that they intended to continue to use VH in their practice, and 97% of respondents agreed or strongly agreed that they would recommend VH to a colleague.

Findings related to providers’ assessment of the impact of VH highlight overwhelming agreement among respondents regarding the benefits of virtual health in healthcare delivery and can be summarized as follows. A significant majority, 95%, indicated that VH increases access to specialist consultation, while 78% recognized its role in improving the quality of in-person referrals. Moreover, 93% acknowledged that VH reduces the time to diagnosis and intervention, demonstrating its efficiency in patient care. Primary care providers expressed strong support, with 98% agreeing that VH enhances their capacity and comfort in managing care plans within the community. Additionally, 99% of respondents recognized VH’s positive impact on patient care in the community. Lastly, 98% agreed that VH facilitates interprofessional communication, collaboration, and learning, underscoring its role in promoting interdisciplinary teamwork and knowledge exchange within healthcare settings.

## Discussion

The current evaluation assessed the utilization, value for money and user experiences associated with the VH platform. This feasibility evaluation has demonstrated success in VH as a viable option for addressing a key priority for the province, namely, enhanced access to specialists through provider-to-provider consultation. The results indicate that providers were using the platform and reported value on many levels from using the platform. We also found that the VH platform offers value for money and high user satisfaction ratings. Our results are consistent with the literature where similar platforms have been reported to improve equitable access to specialist care, helping to address some of the inequities faced by patients [5, 10–12]. There is an anticipation from the preliminary findings that not only will the implementation of VH be sustained, but it will also continue to grow in its utilization across multiple specialties in the province. Notably by the end of February 2024, the number of consults completed had increased from 6,072 reported at the end of January 2024 to 6,535, representing an increase of 463 or 8%. Furthermore, the number of specialists on the platform had increased from 143 to 148, representing an increase of 5 or 3.5%. Similarly, the number of primary care providers using the platform had increased from 873 to 884, representing an increase of 11 or 1.3% for the same period.

The economic analysis demonstrated potential monetary benefits associated with VH. The net savings were still positive in a hypothetical environment where costs doubled. However, we could only quantify some of the benefits related to VH. For instance, if VH consultations result in early diagnosis and treatment, the system could have additional cost savings, which we cannot measure. The consultation and provider experiences were largely positive. The global feedback was to ensure the alignment of VH evolution with the population’s needs. The economic analysis focused on a short-term perspective, as the VH platform is still relatively new in our context. With more specialists joining the platform, assuming no attrition, we can expect to see an increase in the number of referrals avoided, impacting both the monetary benefits and costs. While a future economic analysis will be necessary to examine the long-term impact, our sensitivity analysis, particularly the probabilistic sensitivity analysis, ensures the reported results are robust. The 95% confidence intervals provide a strong range of estimated outcomes, while the analytical approach inspires confidence in the reported results.

Our results build upon a previous study reporting exclusively on the results of the post-consultation survey [3] by adopting a robust evaluation approach based on implementation science principles and expanding on providers’ experiences with individual consultations to describe their overarching experience integrating VH into their clinical practice. Additionally, the economic analysis we conducted to quantify the potential monetary benefit of VH makes a unique contribution to the evidence base on the return on investment associated with the implementation of virtual provider-to-provider consultation platforms.

While offering advantages in terms of convenience and feasibility, the pragmatic sampling in survey design also comes with several weaknesses. Because participants who completed the surveys did so voluntarily, whether the findings from the surveys can be generalized to the larger population is an open question, which could limit the external validity of our findings. The voluntary participation in the surveys also means that the sample may not accurately reflect the population’s diversity, potentially compromising our results’ internal validity. Additionally, we did not conduct a comparative analysis that would have required 95% confidence intervals (95% CI) to understand the range of plausible values for these differences. Furthermore, we also did not test any hypotheses.

Despite these weaknesses, our results are robust, given the different perspectives.

## Conclusions

This feasibility evaluation has demonstrated the value of enabling early access to specialist care through physician-to-specialist phone consults, adding to the existing body of knowledge and making this study highly relevant. VH consultations provided patients easier access to specialist expertise, enabling them to receive timely consultations without travel. This reduces barriers to accessing specialized care and streamlines the referral process by facilitating quick and efficient communication between primary care providers and specialists. By leveraging VH consultations, specialists could efficiently review cases and prioritize consultations, maximizing the impact of their expertise and reducing wait times for patients. VH consultations also fostered collaboration and interdisciplinary communication among healthcare professionals, enabling specialists to offer guidance, share expertise, and provide recommendations to primary care providers, leading to more coordinated and comprehensive patient care. From a cost-effectiveness perspective, VH consultations offered savings by minimizing unnecessary in-person referrals, diagnostic tests, and travel costs associated with in-person specialist visits, benefiting patients and the healthcare system. Our study demonstrates that VH consultations provide numerous benefits that enhance healthcare delivery and reduce costs, making VH an effective approach to healthcare delivery.

## Supplementary Information

Supplementary Material 1. Standards for Reporting Implementation Studies: the StaRI checklist for completion. This file includes a checklist confirming the submission’s adherence to the StaRI reporting guidelines.

Supplementary Material 2. Images of the Virtual Hallway consult booking platform.

Supplementary Material 3. Short post-consultation surveys.

Supplementary Material 4. Provider experience survey.

## Data Availability

The study data is available upon request upon study completion from the corresponding author (TS). The data are not publicly available to protect the privacy of participants.

## Abbreviations

CBA: Cost–benefit analysis
CFIR: Consolidated Framework for Implementation Research
CI: Confidence interval
MSU: Medical service unit
NASSS: Non–adoption, Abandonment, Scale–up, Spread, and Sustainability
NP: Nurse practitioner
PSA: Probability sensitivity analysis
REB: Research Ethics Board
ROI: Return on investment
VH: Virtual Hallway
