# Supplementary material for: Improved access and care through the implementation of virtual Hallway, a consultation platform in Nova Scotia: preliminary findings from a feasibility evaluation
Source: Implement Sci Commun. 2024 Oct 18;5:116. doi: 10.1186/s43058-024-00651-3 (PMC11488209; doi:10.1186/s43058-024-00651-3)
Supplement: Supplementary file 2 — Supplementary Material 2. Images of the Virtual Hallway consult booking platform. [file 43058_2024_651_MOESM2_ESM.pdf]

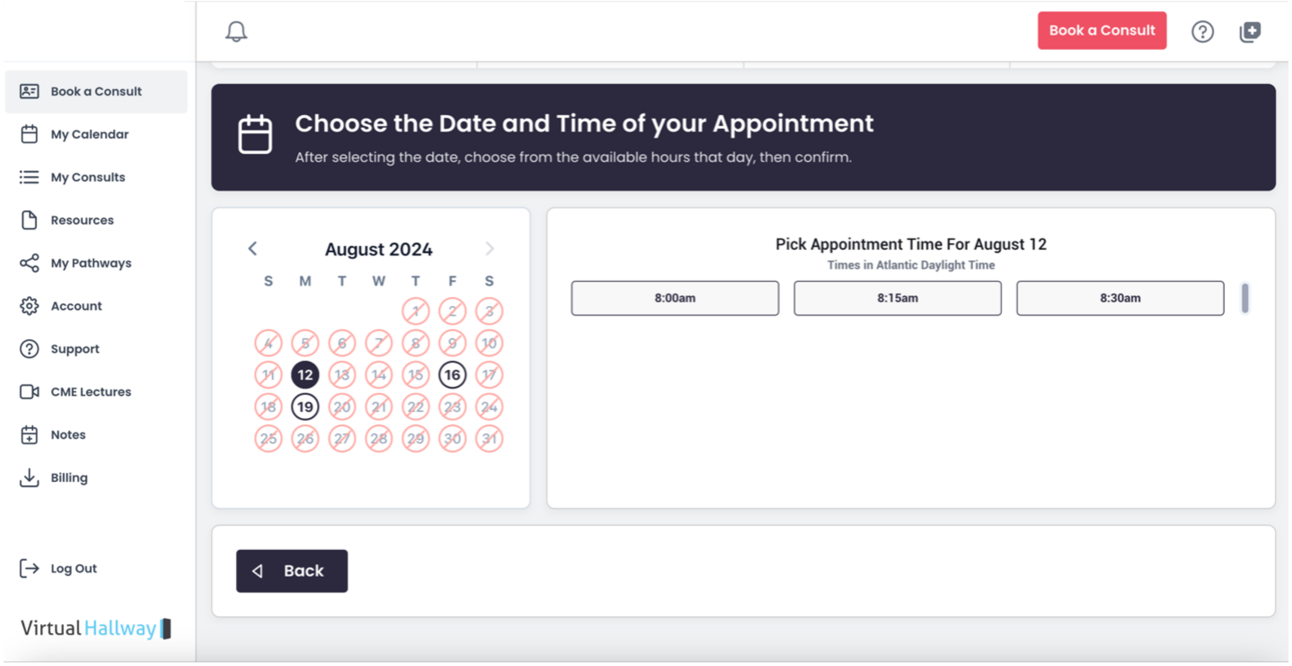
Book a Consult
Book a Consult
My Calendar
My Consults
Resources
My Pathways
Account
Support
CME Lectures
Notes
Billing
Log Out
Virtual Hallway
Choose the Date and Time of your Appointment
After selecting the date, choose from the available hours that day, then confirm.
August 2024
S M T W T F S
1 2 3
4 5 6 7 8 9 10
11 12 13 14 15 16 17
18 19 20 21 22 23 24
25 26 27 28 29 30 31
Pick Appointment Time For August 12
Times in Atlantic Daylight Time
8:00am
8:15am
8:30am
Back

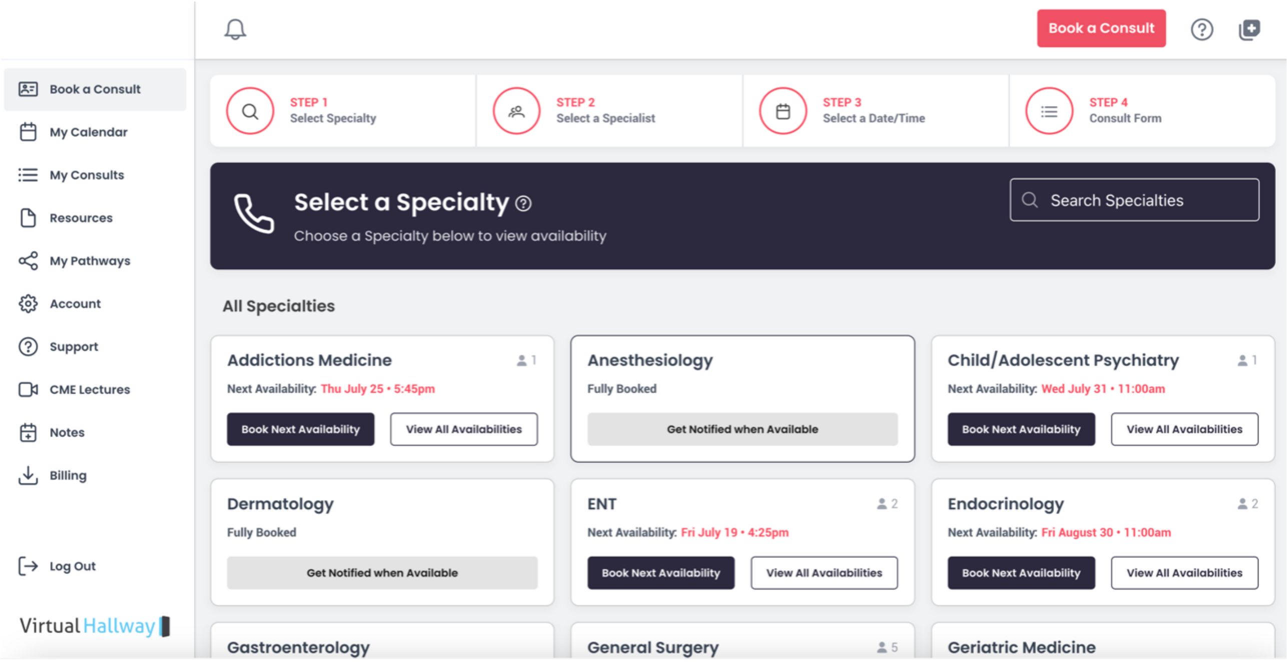
Book a Consult
Book a Consult
My Calendar
My Consults
Resources
My Pathways
Account
Support
CME Lectures
Notes
Billing
Log Out
Virtual Hallway
STEP 1
Select Specialty
STEP 2
Select a Specialist
STEP 3
Select a Date/Time
STEP 4
Consult Form
Select a Specialty
Choose a Specialty below to view availability
Search Specialties
All Specialties
Addictions Medicine
1
Next Availability: Thu July 25 • 5:45pm
Book Next Availability
View All Availabilities
Anesthesiology
Fully Booked
Get Notified when Available
Child/Adolescent Psychiatry
1
Next Availability: Wed July 31 • 11:00am
Book Next Availability
View All Availabilities
Dermatology
Fully Booked
Get Notified when Available
ENT
2
Next Availability: Fri July 19 • 4:25pm
Book Next Availability
View All Availabilities
Endocrinology
2
Next Availability: Fri August 30 • 11:00am
Book Next Availability
View All Availabilities
Gastroenterology
General Surgery
5
Geriatric Medicine
