# Supplementary material for: Improved access and care through the implementation of virtual Hallway, a consultation platform in Nova Scotia: preliminary findings from a feasibility evaluation
Source: Implement Sci Commun. 2024 Oct 18;5:116. doi: 10.1186/s43058-024-00651-3 (PMC11488209; doi:10.1186/s43058-024-00651-3)
Supplement: Supplementary file 3 — Supplementary Material 3. Short post-consultation surveys. [file 43058_2024_651_MOESM3_ESM.pdf]

Consultation Form – Primary Care Provider (For immediate integration into VH Platform)

Please rate your level of satisfaction with your experience using Virtual Hallway for this consultation:

- Very Satisfied
- Satisfied
- Neutral
- Unsatisfied
- Very Unsatisfied

Did this Virtual Hallway consultation avoid the need for a referral? (yes/no)

IF NO – Was this consultation intended to avoid referral? (yes/no)
IF NO – Did this consultation improve the quality of your referral? (yes/no)
IF NO - Did this consultation improve the patient's care while they wait for an in-person referral?

Consultation Form – Specialist (For immediate integration into VH Platform)

Please rate your level of satisfaction with your experience using Virtual Hallway for this consultation:

- Very Satisfied
- Satisfied
- Neutral
- Unsatisfied
- Very Unsatisfied

If this consultation had gone directly to referral, would you have qualified that referral appointment as:

- Necessary
- Somewhat Necessary
- Neutral/Undecided
- Somewhat Unnecessary
- Unnecessary

Endline Provider Experience Survey (For distribution at "endline")

What is your medical specialty? (dropdown of relevant medical specialties)

Please rate your overall level of satisfaction with your experience using Virtual Hallway for consultations:

- Very Satisfied
- Satisfied
- Neutral
- Unsatisfied
- Very Unsatisfied

Please rate your level of agreement with the following statements about your experience using Virtual Hallway: (Matrix with horizontal radio buttons)

The Virtual Hallway platform is easy to use

- Strongly Disagree
- Disagree
- Neutral
- Agree
- Strongly agree

The Virtual Hallway platform's functionality meets my needs

- Strongly Disagree
- Disagree
- Neutral
- Agree
- Strongly agree

The Virtual Hallway platform supports safety and privacy

- Strongly Disagree
- Disagree
- Neutral
- Agree
- Strongly agree

Virtual Hallway is useful for my work

- Strongly Disagree
- Disagree
- Neutral
- Agree
- Strongly agree

Virtual Hallway supports interprofessional communication, collaboration, and learning

- Strongly Disagree
- Disagree
- Neutral
- Agree
- Strongly agree

Virtual Hallway increases access to specialist consultation

- Strongly Disagree
- Disagree
- Neutral
- Agree
- Strongly agree

Virtual Hallway supports primary care provider’s ability to manage care plans in the community

- Strongly Disagree
- Disagree
- Neutral
- Agree
- Strongly agree

Virtual Hallway increases the quality of in-person referrals

- Strongly Disagree
- Disagree
- Neutral
- Agree
- Strongly agree

Access to Virtual Hallway enhances patient care in the community

- Strongly Disagree
- Disagree
- Neutral
- Agree
- Strongly agree

Access to Virtual Hallway reduces time to diagnosis and intervention

- Strongly Disagree
- Disagree
- Neutral
- Agree
- Strongly agree

I intend to continue to use Virtual Hallway in my practice

- Strongly Disagree
- Disagree
- Neutral
- Agree
- Strongly agree

I would recommend using Virtual Hallway to a colleague

- Strongly Disagree
- Disagree
- Neutral
- Agree
- Strongly agree

Please use this space to provide any additional comments about your experience using Virtual Hallway for consultations: (open text)

Would you be interested in participating in a follow-up interview to discuss your experience using Virtual Hallway for consultations? (Yes/No)

If Yes – Please provide your name:
If Yes – Please provide your email address:
If Yes – Please provide your phone number:
