# Supplementary material for: Improved access and care through the implementation of virtual Hallway, a consultation platform in Nova Scotia: preliminary findings from a feasibility evaluation
Source: Implement Sci Commun. 2024 Oct 18;5:116. doi: 10.1186/s43058-024-00651-3 (PMC11488209; doi:10.1186/s43058-024-00651-3)
Supplement: Supplementary file 4 — Supplementary Material 4. Provider experience survey. [file 43058_2024_651_MOESM4_ESM.pdf]

VIRTUAL HALLWAY – PROVIDER EXPERIENCE SURVEY

What is your profession? (drop down – single select)

- Family Physician
- Nurse Practitioner
- Anaesthesiology
- Child/Adolescent Psychiatry
- Endocrinology
- ENT
- Dermatology
- Gastroenterology
- General Surgery
- Hematology
- Hepatology
- Infectious Disease
- Internal Medicine
- OB/GYN
- Orthopedics
- Pain Management
- Pediatric Allergy
- Pediatrics
- Psychiatry
- Rheumatology
- Sleep Medicine
- Urology

In what Nova Scotia Health Zone do you currently practice? (Please select all that apply) (vertical radio buttons – multiselect)

- Central Zone
- Eastern Zone
- Northern Zone
- Western Zone

In what city(ies)/town(s) do you currently practice? (open text)

How long ago did you first use Virtual Hallway? (vertical radio buttons – single select)

- Less than 1 month ago
- 1-3 months ago
- 4-6 months ago
- More than 6 months ago

VIRTUAL HALLWAY – PROVIDER EXPERIENCE SURVEY

How many times have you used Virtual Hallway? (vertical radio buttons – single select)

- Never
- 1-5 times
- 6-10 times
- 10-20 times
- 21+ times

How are you integrating Virtual Hallway into your practice? (vertical radio buttons – multiselect)

- I have a regular time blocked in my schedule to engage in consultations
- I engage in consultations during gaps in my schedule
- I fit consultations into my schedule when needed
- I engage in consultations outside of regular practice hours
- Other (Please specify)

If Profession = Family Physician OR Nurse Practitioner
What types of specialists have you consulted using Virtual Hallway? (Please select all that apply) (vertical radio buttons – multiselect)

- Anaesthesiology
- Child/Adolescent Psychiatry
- Endocrinology
- ENT
- Dermatology
- Gastroenterology
- General Surgery
- Hematology
- Hepatology
- Infectious Disease
- Internal Medicine
- OB/GYN
- Orthopedics
- Pain Management
- Pediatric Allergy
- Pediatrics
- Psychiatry
- Rheumatology
- Sleep Medicine
- Urology

If Profession = Family Physician OR Nurse Practitioner
Please rate your overall level of satisfaction using Virtual Hallway to access specialist consultations (Horizontal radio buttons – single select)

- Very dissatisfied
- Dissatisfied
- Neutral
- Satisfied
- Very Satisfied

If Profession = 'Specialist
Please rate your overall level of satisfaction using Virtual Hallway to offer specialist consultations (Horizontal radio buttons – single select)

- Very dissatisfied
- Dissatisfied
- Neutral
- Satisfied
- Very Satisfied

Please rate your level of agreement with the following statements about your experience using Virtual Hallway: (Horizontal radio buttons – single select)

Virtual Hallway increases access to specialist consultation

- Strongly Disagree
- Disagree
- Neutral
- Agree
- Strongly Agree
- Not Applicable

Virtual Hallway increases the quality of in-person referrals

- Strongly Disagree
- Disagree
- Neutral
- Agree
- Strongly Agree
- Not Applicable

Access to Virtual Hallway reduces time to diagnosis and intervention
- Strongly Disagree
- Disagree
- Neutral
- Agree
- Strongly Agree
- Not Applicable

If Profession = Family Physician OR Nurse Practitioner
Access to Virtual Hallway increases my capacity to, and comfort in, managing care plans in the community
- Strongly Disagree
- Disagree
- Neutral
- Agree
- Strongly Agree
- Not Applicable

Access to Virtual Hallway enhances patient care in the community
- Strongly Disagree
- Disagree
- Neutral
- Agree
- Strongly Agree
- Not Applicable

Virtual Hallway supports interprofessional communication, collaboration, and learning
- Strongly Disagree
- Disagree
- Neutral
- Agree
- Strongly Agree
- Not Applicable

Virtual Hallway is easy to use
- Strongly Disagree
- Disagree
- Neutral
- Agree
- Strongly Agree
- Not Applicable

VIRTUAL HALLWAY – PROVIDER EXPERIENCE SURVEY

The functionality of the Virtual Hallway platform meets my practice needs
- Strongly Disagree
- Disagree
- Neutral
- Agree
- Strongly Agree
- Not Applicable

Virtual Hallway is a safe and private platform
- Strongly Disagree
- Disagree
- Neutral
- Agree
- Strongly Agree
- Not Applicable

Virtual Hallway is useful in my practice
- Strongly Disagree
- Disagree
- Neutral
- Agree
- Strongly Agree
- Not Applicable

I intend to continue to use Virtual Hallway in my practice
- Strongly Disagree
- Disagree
- Neutral
- Agree
- Strongly Agree
- Not Applicable

I would recommend using Virtual Hallway to a colleague
- Strongly Disagree
- Disagree
- Neutral
- Agree
- Strongly Agree
- Not Applicable

Please use the space below to provide any additional feedback about your experience using Virtual Hallway: (open text box)

Would you be interested in participating in an interview with the Implementation Science Team at Nova Scotia Health about your experience using Virtual Hallway? (vertical radio buttons – single select)

- Yes
- No

If YES
Please provide your name: (open text)

If YES
Please provide your phone number: (open text)

If YES
Please provide your email address: (open text)
